# Base excision repair in *Staphylococcus aureus*: a defense against oxidative DNA damage and a potential target for novel antibiotics

**DOI:** 10.3389/fmicb.2026.1887239

**Published:** 2026-08-17

**Authors:** Aigerim Turgimbayeva, Bakhtiyar Yakupov, Sailau Abeldenov

**Affiliations:** National Center for Biotechnology, Astana, Kazakhstan

**Keywords:** antibiotic resistance, antibiotic target, base excision repair, novel antibiotics, oxidative DNA damage, oxidative stress, *Staphylococcus aureus*

## Abstract

*Staphylococcus aureus* (*S. aureus*) remains a major clinical challenge due to its high virulence, persistence, and remarkable capacity to develop antibiotic resistance. Survival within the host requires adaptation to oxidative and nitrosative stress generated by immune cells, which inflict extensive damage on bacterial DNA, including oxidized bases, abasic sites, and strand breaks. Under these conditions, the base excision repair (BER) pathway plays a central role in maintaining genome integrity and supporting bacterial survival under immune and antibiotic-induced stress. This review examines the organization and functional significance of BER in *S. aureus*, with particular emphasis on its contribution to oxidative stress tolerance, genome stability, and adaptive responses relevant to antimicrobial resistance. Comparative analysis of BER components in *S. aureus* and *Escherichia coli* (*E. coli*) highlights both conserved and species-specific features, reflecting differences in ecological adaptation, stress response strategies, and functional redundancy within DNA repair networks. Notably, the apparently lower redundancy of several BER-associated enzymes in *S. aureus* may create exploitable vulnerabilities for antimicrobial intervention. The therapeutic targeting of BER represents a promising but complex strategy. Inhibition of selected BER-associated enzymes may enhance antibiotic efficacy by promoting the accumulation of oxidative DNA damage and toxic repair intermediates. However, disruption of DNA repair pathways may also increase mutagenesis and accelerate adaptive evolution, potentially contributing to resistance development. Understanding the dual role of BER as both a protective mechanism and a potential source of vulnerability is therefore essential for the rational design of novel antibacterial approaches. This review highlights BER as a promising source of antibacterial targets while emphasizing that individual BER components differ substantially in their biological importance, functional redundancy, druggability and level of experimental validation. Further genetic, biochemical and pharmacological studies will be required to translate BER-targeted strategies into effective therapies against *S. aureus*.

## 1 Introduction

*S. aureus* is a major opportunistic pathogen that colonizes the anterior nares, skin and pharynx of approximately 25% of healthy individuals and causes a wide spectrum of diseases, ranging from skin and soft tissue infections to life-threatening invasive infections, including bacteremia, infective endocarditis, osteomyelitis, pneumonia, toxic shock syndrome and abscess formation (Coll et al., 2025; Taylor et al., 2025). *S. aureus* bacteremia is associated with high mortality, and *S. aureus* remains one of the leading bacterial causes of death worldwide (Tong et al., 2025). The World Health Organization identified antimicrobial resistance as one of the major threats to global health, with antibiotic-resistant *S. aureus* remaining among the priority pathogens requiring new therapeutic strategies (World Health Organization, 2025). The continued emergence and dissemination of antibiotic-resistant strains, including methicillin-resistant *Staphylococcus aureus* (MRSA), has intensified the need to identify bacterial pathways that can be exploited for novel or adjunctive antimicrobial strategies. Consequently, bacterial DNA damage response and repair pathways have emerged as attractive complementary therapeutic targets because they contribute to bacterial survival under host-induced stress while influencing persistence, genome stability, mutagenesis, and the evolution of antibiotic resistance.

Current antibacterial agents predominantly target a limited number of essential bacterial processes, including cell wall biosynthesis, protein synthesis, DNA replication, transcription, and the maintenance of DNA topology (Darby et al., 2023). In contrast, DNA repair pathways have historically received considerably less attention as antibacterial targets despite their fundamental role in maintaining genome integrity under stressful conditions. Increasing evidence indicates that DNA repair systems contribute not only to bacterial survival during infection but also to persistence, adaptive mutagenesis, and the evolution of antibiotic resistance, making them attractive candidates for adjunctive antimicrobial strategies.

During infection, *S. aureus* is continuously exposed to oxidative and nitrosative stress generated by neutrophils and macrophages as part of the host innate immune response (Miller and Britigan, 1997; Rigby and DeLeo, 2012). Reactive oxygen and nitrogen species damage multiple cellular components, including DNA, RNA, proteins and lipids, thereby threatening bacterial viability. DNA is particularly susceptible to oxidation, deamination, alkylation, and strand break formation, resulting in a broad spectrum of lesions that require efficient repair to maintain genome stability.

To counteract these lesions, *S. aureus*, like other bacteria, employs several DNA repair pathways, including base excision repair (BER), nucleotide excision repair (NER), mismatch repair (MMR), homologous recombination (HR), and the SOS response. Among these pathways, BER represents the principal mechanism responsible for repairing oxidative and many nitrosative DNA lesions (van der Veen and Tang, 2015).

Despite the growing interest in bacterial DNA repair, many aspects of BER in *S. aureus* remain poorly understood, and much of the current mechanistic knowledge continues to be extrapolated from model organisms such as *E. coli*.

This review critically examines the current understanding of BER in *S. aureus*, with particular emphasis on its role in protecting the pathogen from host-induced oxidative and nitrosative DNA damage. Particular attention is given to experimentally validated findings in *S. aureus*, while evidence from other bacterial species is discussed only where it provides important mechanistic context. We further examine the organization of BER, its interplay with other DNA repair pathways, and critically assess both the opportunities and current limitations of BER-targeted antimicrobial strategies. Finally, we identify key knowledge gaps and future research priorities.

## 2 Host-induced oxidative and nitrosative stress during *S. aureus* infection

### 2.1 Host immune pressure creates oxidative and nitrosative stress

*S. aureus* is capable of long-term asymptomatic colonization but may also cause invasive infections following disruption of epithelial barriers or impairment of host defenses. Upon invasion of host tissues, bacterial products and tissue damage activate the innate immune response, leading to rapid recruitment of neutrophils and macrophages (Novick, 2003). Among these immune cells, neutrophils represent the primary defense against *S. aureus*, as demonstrated by the markedly increased susceptibility to invasive staphylococcal infections in patients with impaired neutrophil function, including those with chronic granulomatous disease (Buvelot et al., 2017).

Following phagocytosis, two principal outcomes are possible. In the first, neutrophils efficiently eliminate engulfed bacteria and subsequently undergo apoptosis, followed by macrophage-mediated efferocytosis. Alternatively, *S. aureus* may evade killing through the production of pore-forming toxins, including hemolysins and leukocidins, which promote neutrophil lysis and facilitate bacterial dissemination (Rigby and DeLeo, 2012; Guerra et al., 2017).

### 2.2 Oxidative and nitrosative burst

During phagocytosis, activation of the innate immune response triggers a rapid and robust production of reactive oxygen species (ROS) and reactive nitrogen species (RNS). Within the phagosome, the NADPH oxidase complex generates superoxide (${\text{O}}_{2^{•}}$−), which undergoes spontaneous or enzymatic dismutation to hydrogen peroxide (H_2_O_2_). Secondary metal-catalyzed reactions may generate hydroxyl radicals (•OH), whereas neutrophil myeloperoxidase uses H_2_O_2_ and chloride ions to produce hypochlorous acid/hypochlorite (HOCl/OCl^−^). In parallel, inducible nitric oxide synthase (iNOS) in macrophages produces nitric oxide (•NO), which reacts with superoxide to form peroxynitrite (ONOO^−^). These reactive species create a strongly oxidative and nitrosative environment within the phagosome and contribute substantially to the killing of *S. aureus* (Fialkow et al., 2007; Guerra et al., 2017).

Although ROS and RNS damage proteins, lipids and RNA, DNA represents one of the most vulnerable intracellular targets because accumulated lesions directly compromise genome stability, DNA replication and bacterial viability.

### 2.3 Biological consequences of oxidative DNA damage

Oxidative and nitrosative stress induces a broad spectrum of DNA lesions, including oxidized bases, abasic sites, deamination products, DNA-protein crosslinks, and single- or double-strand breaks (Sung et al., 2013). Hydroxyl radicals generate oxidized purines and pyrimidines, including 8-oxoG, FapyA, FapyG and 5-hydroxycytosine, whereas peroxynitrite promotes DNA oxidation and cytosine deamination, resulting in uracil formation and additional mutagenic lesions. If left unrepaired, these lesions impair DNA replication, alter gene expression, increase mutagenesis and ultimately reduce bacterial fitness.

To preserve genome integrity under these conditions, *S. aureus* relies on coordinated DNA repair pathways, among which BER constitutes the primary mechanism responsible for repairing oxidized and deaminated bases, abasic sites and many other oxidative DNA lesions (van der Veen and Tang, 2015).

**Figure 1:**
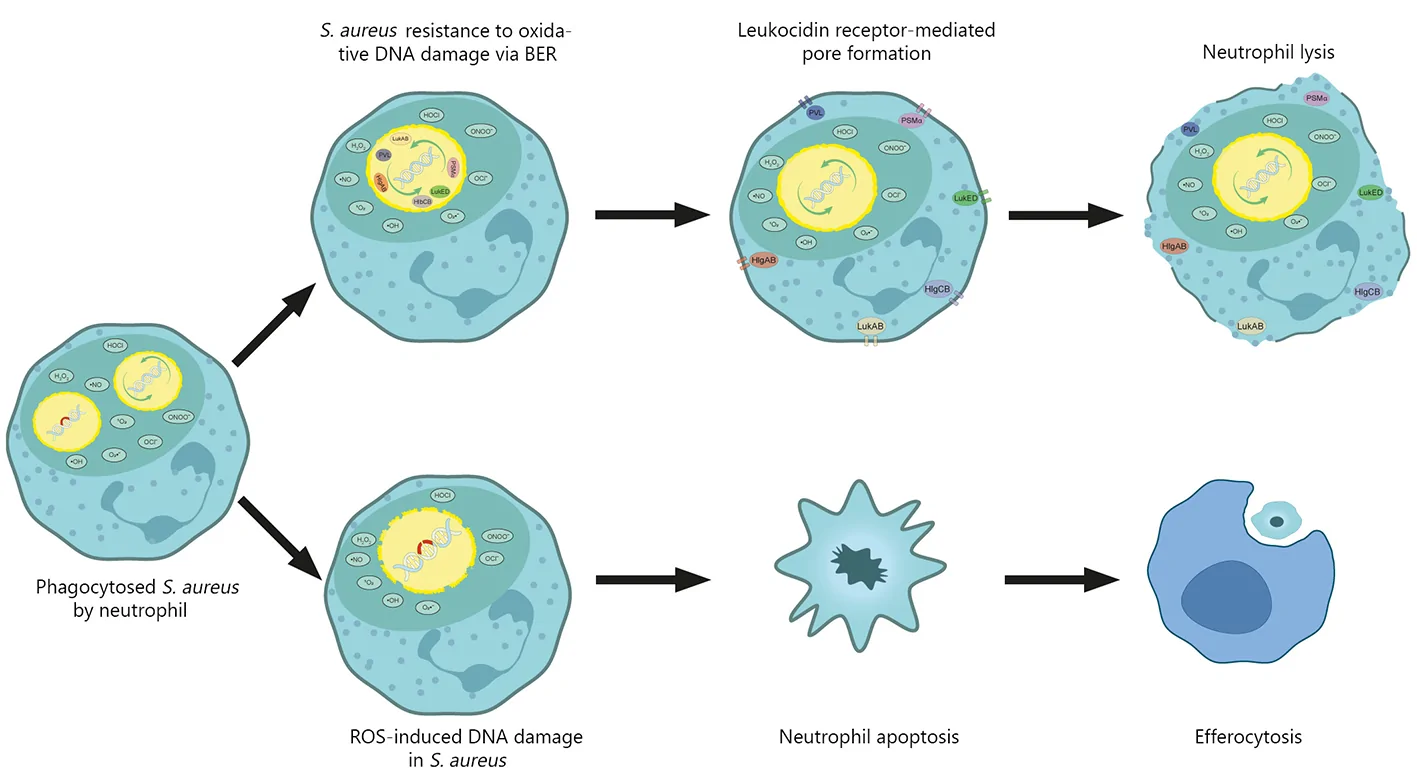
Conceptual model of the interaction between host-induced oxidative stress, bacterial genome maintenance and neutrophil fate during *S. aureus* infection. Following phagocytosis, ROS and RNS may damage bacterial DNA and reduce bacterial fitness. Efficient DNA repair, including BER, may support survival under these conditions, whereas cytolytic toxins independently contribute to neutrophil injury and lysis. The proposed connection between BER activity and sustained virulence-factor expression remains hypothetical and requires direct experimental validation.

Because BER repairs oxidative DNA lesions, it is expected to contribute to genome maintenance during exposure to host-derived oxidative and nitrosative stress. BER may therefore support bacterial fitness under infection-associated stress, although its specific contribution to bacterial survival and persistence during infection has not yet been directly demonstrated in *S. aureus* (Figure 1).

Thus, oxidative and nitrosative stress generated by host phagocytes creates one of the principal selective pressures encountered by *S. aureus* during infection. Survival under these conditions depends not only on antioxidant defense systems but also on efficient repair of oxidative DNA damage. Given its central role in repairing oxidative DNA damage in bacteria, BER has been proposed as a promising target for antimicrobial intervention (Kurthkoti et al., 2020). However, further studies are required to establish the contribution of BER to the pathogenesis and persistence of *S. aureus* during infection.

## 3 Oxidative and nitrosative DNA lesions and their repair pathways

### 3.1 Oxidative and nitrosative DNA lesions

ROS and RNS generate chemically distinct DNA lesions that differ in their origin, mutagenic potential and repair requirements (Sung et al., 2013). The hydroxyl radical induces oxidative damage to DNA bases (7, 8-dihydro-8-oxoguanine (8-oxoG), 5-hydroxycytosine, FapyA, and FapyG), and also leads to the formation of AP sites and single-strand breaks. Spatial clustering of such lesions can subsequently result in the formation of DNA double-strand breaks. Peroxynitrite causes nitrosylation and deamination (cytosine to uracil), further increasing the mutational burden. In addition to deamination reactions, peroxynitrite oxidizes DNA and preferentially reacts with guanine, forming the most common purine lesion, 8-oxoG. 8-oxoG is capable of mispairing with adenine, thereby inducing G:C → T:A transversions. BER plays a primary role in the removal of mentioned lesions (van der Veen and Tang, 2015). Thus, ROS and RNS induce a wide spectrum of DNA damage, predominantly represented by oxidized bases and the formation of AP sites. These lesions are repaired by BER, which plays a central role in maintaining genomic stability and bacterial viability.

### 3.2 DNA repair pathways responding to oxidative DNA damage

#### 3.2.1 Base excision repair (BER)

BER is the principal pathway responsible for repairing small, non-bulky DNA lesions generated by oxidation, deamination and alkylation. BER is initiated by DNA glycosylases that recognize and excise damaged bases, generating abasic (AP) sites that are subsequently processed by AP endonucleases, DNA polymerases and DNA ligases.

In bacteria, BER is initiated by a DNA glycosylase, which hydrolyses the N-glycosidic bond between the damaged base and the sugar moiety. DNA glycosylases are classified as either monofunctional or bifunctional. Monofunctional DNA glycosylases produce AP sites, which are then cleaved by an AP endonuclease, generating 3′-OH and 5′-deoxyribose phosphate (5′-dRP) ends. The 5′-dRP group is removed by a dRP lyase, resulting in a 5′-phosphate end. Bifunctional DNA glycosylases cut the AP site to form a 3-α,β-unsaturated aldehyde (3′-PA) and a 5′-phosphate terminus. Some bifunctional DNA glycosylases (such as Fpg and Nei) can modify the 3-α,β-unsaturated aldehyde further through δ-elimination, creating a 3′-phosphate end.

AP endonucleases supply the free 3′-OH and 5′-phosphate ends needed for the functions of DNA polymerase and DNA ligase, respectively. DNA synthesis and ligation then occur via one of two pathways: short-patch BER, which involves inserting a single nucleotide, or long-patch BER, which involves adding between two and eight nucleotides. In *E. coli*, BER mainly proceeds through the short-patch pathway under normal physiological conditions, whereas in *Mycobacterium tuberculosis*, long-patch repair is more prevalent (Dianov and Lindahl, 1994; Kumar et al., 2011). The type of DNA glycosylase and the character of the DNA lesion can influence the choice of repair pathway. Short-patch repair involves removing a single damaged nucleotide and replacing it with the correctly paired base. In *E. coli*, DNA polymerase *I* performs the insertion of the new nucleotide, after which DNA ligase LigA seals the nick. Long-patch repair starts with removing the damaged residue and then synthesizes several nucleotides. DNA polymerase *I* uses the 3′-OH end created by AP endonuclease cleavage of the phosphodiester bond to synthesize the replacement strand. During this process, the parental strand with the 5′-dRP is displaced, forming a flap structure, which is then cleaved by the 5′ → 3′ exonuclease activity of the same polymerase. The process concludes with LigA, which ligates the ends to complete the repair (Kurthkoti et al., 2020).

One of the best-characterized BER-associated systems in bacteria is the GO system, which protects cells from the mutagenic effects of (8-oxoG), one of the most frequent and promutagenic oxidative DNA lesions. The canonical bacterial GO system includes MutT, MutM/Fpg and MutY. MutT sanitizes the nucleotide pool by hydrolyzing 8-oxo-dGTP before its incorporation into DNA; MutM/Fpg excises 8-oxoG from 8-oxoG:C pairs; and MutY removes adenine misincorporated opposite 8-oxoG, thereby preventing fixation of G:C → T:A transversions. In *S. aureus*, disruption of the GO-system glycosylases MutM and MutY has been shown to increase spontaneous mutation frequency and generate mutator phenotypes (Canfield et al., 2013), thereby enhancing the adaptive potential of bacterial populations. However, a clearly defined MutT-like functional counterpart in *S. aureus* remains less certain (Canfield et al., 2013).

#### 3.2.2 Nucleotide excision repair (NER)

Bacterial NER, mediated by UvrABC, primarily recognizes helix-distorting lesions rather than specific chemical modifications. Therefore, NER is most important for bulky DNA adducts, UV-induced photoproducts and some crosslinks. Its contribution to oxidative DNA damage repair is more limited and lesion-dependent. Some oxidative lesions, such as thymine glycol, can be recognized by UvrABC in biochemical systems, but most small oxidized bases are primarily repaired by BER (Kow et al., 1990). Importantly, a genome-wide Tn-seq analysis in *S. aureus* identified *uvrA* and *uvrC* among the genes required for fitness during nitric oxide stress. Targeted deletion of *uvrAB* or *uvrC* resulted in prolonged lag phases and impaired growth in the presence of nitric oxide, providing direct evidence that NER contributes to nitrosative stress resistance in this pathogen (Grosser et al., 2018).

#### 3.2.3 Mismatch repair (MMR)

MMR primarily corrects replication errors, but it can also contribute indirectly to the control of oxidative mutagenesis by recognizing mismatches generated when oxidized bases are bypassed during replication. In *E. coli*, the MutHLS system uses Dam methylation for strand discrimination. This paradigm does not fully apply to many Gram-positive bacteria, including *S. aureus*, which lack MutH homologs. *S. aureus* encodes MutS and MutL homologs, and direct experimental evidence confirms their importance in mutation control. Insertional inactivation of *mutS* in strain RN4220 increased the frequencies of resistance mutations to rifampicin, fusidic acid, norfloxacin and mupirocin by up to 78-fold. However, screening of 493 clinical isolates did not identify strains with a ≥10-fold increase in mutation frequency, suggesting that stable high-level mutator phenotypes may be relatively uncommon among clinical *S. aureus* populations (O'Neill and Chopra, 2002). Disruption of *mutS* and *mutL* has been clearly associated with hypermutability in *S. aureus*, confirmed by complementation analysis (Prunier and Leclercq, 2005). Nevertheless, the precise mechanism of strand discrimination and the contribution of MMR to oxidative mismatch repair remain less well defined than in *E. coli*. This distinction is important because mechanistic assumptions based on *E. coli* cannot be directly transferred to *S. aureus*.

#### 3.2.4 Homologous recombination (HR)

HR is not a direct repair pathway for oxidized bases. Instead, it repairs double-strand breaks that may arise when replication forks encounter unrepaired oxidative lesions or clustered DNA damage. In Gram-positive bacteria, including *S. aureus*, AddAB performs functions analogous to RecBCD in processing DNA ends for RecA-mediated repair. Thus, the relationship between HR and oxidative stress is indirect: oxidative lesions can generate replication-associated breaks, whereas HR repairs the resulting chromosome damage.

#### 3.2.5 SOS response

The SOS response is a regulatory DNA damage-response program rather than a biochemical lesion-removal pathway. It is activated when RecA nucleoprotein filaments stimulate LexA autocleavage, leading to induction of genes involved in DNA repair, damage tolerance and stress survival. Its role is dual: SOS activation can promote survival after genotoxic stress, but it may also increase mutagenesis through error-prone polymerases and thereby contribute to antibiotic resistance evolution. For this reason, RecA and LexA have been considered potential antimicrobial adjuvant targets, especially in strategies aimed at limiting stress-induced mutagenesis rather than directly killing bacteria. Oxidative DNA damage may activate the SOS response indirectly when unrepaired lesions stall replication forks or generate single-stranded DNA regions that promote RecA nucleoprotein filament formation (Gaupp et al., 2012; Gupta and Imlay, 2022). In *S. aureus*, phagocyte-associated oxidative stress has been linked to SOS induction (Cheng et al., 2024). SOS activation promotes DNA damage tolerance and mutagenesis and has been associated, in *S. aureus* and other bacterial contexts, with antibiotic-resistance evolution and persistence-related phenotypes. However, the extent to which it directly controls small-colony variant formation and virulence in *S. aureus* remains incompletely defined (Podlesek and Žgur Bertok, 2020). Consequently, the SOS response represents both a survival mechanism and a potential therapeutic target.

Although several DNA repair and damage-response pathways contribute to genome maintenance, BER remains the primary mechanism for removing most small oxidative and many nitrosative DNA lesions. NER, MMR, HR and SOS function mainly as complementary or downstream systems that repair specific lesion classes, correct replication-associated mismatches, rescue broken replication forks or coordinate broader DNA damage responses.

## 4 Biological significance of BER in *S. aureus*

Unlike many environmental bacteria, *S. aureus* spends a substantial part of its life cycle under continuous oxidative and nitrosative pressure imposed by host immune cells. Under these conditions, maintenance of genome integrity becomes essential not only for bacterial survival but also for sustained expression of virulence determinants.

The outcome of the interaction between neutrophils and *S. aureus* depends on the balance between host oxidative defense mechanisms and bacterial virulence determinants.

Accumulation of oxidative DNA damage compromises genome integrity, limiting the expression of essential survival genes that are normally upregulated during phagocytosis, ultimately resulting in bacterial degradation, neutrophil apoptosis, and subsequent efferocytosis of neutrophils by macrophages (Cohen et al., 2016).

In contrast, during immune evasion, preservation of an intact staphylococcal genome permits the expression of potent cytolytic factors, including leukocidins (LukAB, HlgAB, HlgCB, LukED, and PVL) and phenol-soluble modulins (PSMα), which promote neutrophil lysis (Surewaard et al., 2013; McGuinness et al., 2016).

BER is the major pathway responsible for repairing oxidative DNA base lesions in bacteria (van der Veen and Tang, 2015) and is therefore expected to contribute to genome maintenance in *S. aureus* during exposure to host-derived oxidative stress. Preservation of genome integrity by BER may support bacterial persistence and sustained expression of virulence determinants during infection. However, the specific contribution of BER to these processes has not yet been directly demonstrated in *S. aureus*.

## 5 BER as a target for antimicrobial intervention

Growing evidence indicates that DNA repair plays a central role in the survival of *S. aureus* within host tissues by maintaining genome stability under oxidative and nitrosative stress generated during infection. BER is therefore increasingly considered a potential target for adjunctive antimicrobial strategies aimed at sensitizing bacteria to host immune defenses and conventional antibiotics (Kurthkoti et al., 2020; Ha and Edwards, 2021). The broader feasibility of DNA repair-directed antimicrobial strategies is supported by studies showing that inhibition of the RecA-dependent SOS response reduces resistance emergence and restores susceptibility to subclinical concentrations of ciprofloxacin, whereas disruption of AddAB-mediated double-strand break repair increases the sensitivity of *S. aureus* to fluoroquinolones (Lim et al., 2019; Bradbury et al., 2024). Although RecA and LexA are not BER proteins, inhibition of the RecA-LexA regulatory axis suppresses stress-induced mutagenesis and potentiates antibiotic activity, providing proof of principle that bacterial DNA damage responses can be therapeutically exploited (Wigle et al., 2009; Alam et al., 2016; Diaz-Diaz et al., 2022; Jaramillo et al., 2022; Maso et al., 2022; Schuurs et al., 2023; Chatterjee et al., 2024). However, not all BER components represent equally attractive therapeutic targets. Whereas inhibition of some enzymes primarily increases mutation rates without substantially affecting bacterial viability, others appear to be essential for processing toxic repair intermediates and maintaining genome integrity. Therefore, the therapeutic potential of individual BER proteins should be evaluated according to their biological function, functional redundancy, and experimental validation.

### 5.1 DNA glycosylases: important for genome stability but challenging bactericidal targets

Oxidative stress generates numerous modified bases, among which 8-oxoG is one of the most frequent and mutagenic lesions. In *E. coli*, MutM (Fpg) removes 8-oxoG before DNA replication, whereas MutY excises adenine misincorporated opposite 8-oxoG, thereby preventing G:C → T:A transversions (Michaels et al., 1991, 1992). In *S. aureus*, exposure to nitric oxide increases mutation frequency approximately fivefold in the wild type, whereas Δ*mutY* mutants exhibit an even greater increase in mutagenesis. Complementation restores mutation frequencies to baseline levels, confirming the critical role of MutY in suppressing NO^·^-induced mutagenesis (Hurley et al., 2023).

Most *S. aureus* strains contain single homologs of MutM (SauFpg1) and MutY (SauMutY). More divergent MRSA isolates may encode an additional distant MutM ortholog (SauFpg2), although multiple substitutions within the zinc-finger domain and lesion-recognition loop suggest that its catalytic properties differ from those of SauFpg1 (Endutkin et al., 2021). Similarly, although several MutT-like proteins have been identified genetically, none reproduces the classical *mutT* hypermutator phenotype, and a definitive functional MutT counterpart has not yet been established in *S. aureus* (Canfield et al., 2013; Snell et al., 2021).

Because *S. aureus* lacks a Nei homolog but retains Nth, Nth is likely to represent its principal glycosylase for several oxidized pyrimidines, including thymine glycol (Ambur et al., 2009). Although direct *in vivo* evidence in *S. aureus* remains limited, studies in *Campylobacter jejuni*, which shares the same Nth-positive/Nei-negative organization, demonstrate that Nth deficiency increases the frequency of spontaneous fluoroquinolone-resistant mutants and is associated with mutations in *perR* and *gyrA* (Dai et al., 2019). Nth activity also depends on the Fe-S cluster biogenesis pathway, linking oxidative stress adaptation with BER efficiency (Roberts et al., 2017).

Together, these findings indicate that DNA glycosylases make important contributions to the suppression of oxidative mutagenesis and the maintenance of genome stability. However, inhibition of MutY, MutM or Nth primarily promotes hypermutability rather than bacterial death. Consequently, although these enzymes are biologically important, their therapeutic inhibition could potentially accelerate adaptive evolution and antibiotic resistance, limiting their attractiveness as standalone antibacterial targets.

### 5.2 Uracil-DNA glycosylase (UDG)

Uracil arises in DNA either through cytosine deamination or misincorporation of dUTP during replication. If unrepaired, uracil-containing DNA results in C:G → T:A transition mutations. Uracil-DNA glycosylase (UDG) initiates BER by removing uracil from both U:A and U:G base pairs, thereby preventing fixation of these mutations (Ambur et al., 2009; Zharkov et al., 2010).

An additional feature distinguishing UDG from other BER enzymes is the existence of naturally occurring protein inhibitors, including UGI, p56 and SAUGI, which function by mimicking the negatively charged DNA duplex (Savva and Pearl, 1995; Serrano-Heras et al., 2007; Wang et al., 2014). In *S. aureus*, SAUGI, together with dUTPase and the Stl regulatory protein, forms a complex uracil metabolism network whose physiological roles remain incompletely understood (Papp-Kádár et al., 2019). Because UDG contributes to genome stability and may participate in antiphage defense, its selective inhibition could potentially impair DNA repair while increasing susceptibility to phage infection. Nevertheless, this concept remains largely hypothetical and requires experimental validation *in vivo*. Unlike MutM, MutY and Nth, UDG is supported by the existence of natural protein inhibitors, providing proof that the enzyme can be selectively blocked. However, whether UDG inhibition produces bactericidal effects, increases phage susceptibility, or primarily enhances mutagenesis in *S. aureus* remains unknown.

### 5.3 AP endonuclease Nfo: a potentially vulnerable core BER component

AP sites and blocked 3′ DNA termini generated during oxidative stress must be processed before DNA synthesis can proceed. In *E. coli*, this function is shared by Xth and Nfo, whereas *S. aureus* lacks an Xth homolog and encodes Nfo as its canonical endonuclease IV-type AP endonuclease (Ambur et al., 2009; Turgimbayeva et al., 2022). The absence of a redundant Xth homolog suggests that Nfo occupies a particularly important position in BER. In *E. coli*, Xth provides most AP endonuclease activity, whereas Nfo serves primarily as an auxiliary enzyme that becomes particularly important under oxidative stress conditions (Cunningham et al., 1986; Levin and Demple, 1996). The absence of an Xth counterpart in *S. aureus* may therefore increase functional dependence on Nfo.

AP sites and blocked 3′ termini are potentially cytotoxic BER intermediates because they prevent subsequent DNA synthesis and ligation (Boiteux and Guillet, 2004). Evidence from *E. coli* indicates that disruption of AP-end processing markedly increases susceptibility to oxidative and alkylating agents, supporting the view that downstream processing of BER intermediates may be more important for bacterial viability than loss of individual lesion-specific glycosylases (Cunningham et al., 1986; Harrison et al., 2006).

Biochemical and structural studies support an important role for SaNfo in the processing of oxidative DNA damage and demonstrate its interaction with the DNA sliding β-clamp (Turgimbayeva et al., 2022; Zein et al., 2024). This interaction may coordinate AP-site processing with downstream DNA synthesis. Structural analyses revealed a catalytic center containing two Fe^2+^ and one Zn^2+^ ions, distinct from the three-Zn^2+^ active site of *E. coli* Nfo (Hosfield et al., 1999; Kirillov et al., 2025). Based on these structural data, molecular docking predicted that two peptides, DVLINAYR and APQLLYIVK, may interact with the SaNfo catalytic pocket (Ma et al., 2026). Importantly, no close human structural homolog of bacterial Nfo has been identified (Friedberg et al., 2006), further supporting its therapeutic attractiveness.

Nevertheless, current evidence remains preliminary. Candidate inhibitors have been identified primarily through structural modeling and docking, whereas their antibacterial efficacy, selectivity and *in vivo* activity remain to be demonstrated.

### 5.4 Downstream and multifunctional BER-associated enzymes

Following removal of damaged bases, downstream and multifunctional DNA metabolism enzymes restore DNA integrity through end processing, DNA synthesis and ligation. In *S. aureus*, RecJ participates in DNA end processing during BER, homologous recombination and replication-fork recovery and exhibits structural adaptations associated with stress tolerance (Gao et al., 2026). However, because RecJ functions in several DNA repair pathways, its biological importance does not necessarily establish it as a BER-specific or readily druggable target. Direct evidence that RecJ inhibition is bactericidal or selectively sensitizes *S. aureus* remains lacking. DNA polymerase *I* (PolA) performs gap filling during short- and long-patch BER. In the related Gram-positive bacterium *Streptococcus pyogenes*, PolA1 inactivation does not impair basal growth but reduces DNA repair efficiency and increases sensitivity to H_2_O_2_, suggesting a stress-dependent rather than universally essential role (Toukoki and Gryllos, 2013). Comparable functional validation in *S. aureus* remains limited. PolX provides additional proofreading and may contribute to the removal of misincorporated oxidized nucleotides and ribonucleotides, thereby supporting genome stability (Baños et al., 2010; Nagpal and Nair, 2021; Tanneur et al., 2025). However, its essentiality, target vulnerability and therapeutic selectivity in *S. aureus* have not yet been established.

LigA represents one of the strongest BER-associated DNA metabolism targets, although its essentiality reflects broader functions in DNA replication, recombination and repair rather than a BER-specific role. As an essential NAD^+^-dependent enzyme, bacterial LigA is structurally distinct from human ATP-dependent DNA ligases, providing a basis for selective inhibition. Structural and biochemical characterization of *S. aureus* LigA has identified conserved adenylation and DNA-binding domains and a bacterial-specific NAD^+^-binding architecture, while several small-molecule inhibitors of bacterial NAD^+^-dependent ligases have demonstrated antibacterial activity (Kaczmarek et al., 2001; Meier et al., 2008; Han et al., 2009). Thus, LigA may be more appropriately regarded as a broadly essential bacterial DNA metabolism target that also participates in BER, rather than as a BER-specific vulnerability.

### 5.5 BER-targeted therapy: opportunities and limitations

Experimental evidence demonstrates that BER contributes substantially to the maintenance of genome stability in *S. aureus* under basal and nitrosative stress conditions. Transposon mutants lacking *nth, nfo, ung*, or *mutY* exhibited significantly higher basal mutation frequencies than wild-type *S. aureus*. Exposure to nitric oxide further increased mutagenesis, with a particularly pronounced effect in the Δ*mutY* mutant, while complementation restored mutation frequency toward the wild-type level (Hurley et al., 2023). Coordinated activity of BER together with recombinational repair pathways involving RecG, RecJ and PolA enables adaptation to nitrosative stress within host immune cells (Hurley et al., 2023).

Recent studies have further shown that bacterial lethality under certain physiological conditions may result not simply from ROS accumulation but from the formation of toxic BER intermediates when downstream repair becomes unbalanced (Gruber et al., 2021). The contribution of ROS to antibiotic-mediated bacterial killing remains debated and appears to depend on the antibiotic, bacterial species, metabolic state and experimental conditions. ROS should therefore not be regarded as a universal downstream mechanism of bactericidal antibiotic action. Instead, the available evidence supports a context-dependent model in which oxidative stress may amplify lethality when DNA repair and cellular homeostasis are already disrupted. These observations indicate that BER functions in a highly context-dependent manner, and therapeutic outcomes may depend on bacterial physiology as much as on inhibition of individual repair enzymes.

Overall, BER provides several conceptually distinct opportunities for antimicrobial intervention, but the therapeutic potential of individual components differs substantially. DNA glycosylases such as MutY, MutM and Nth primarily suppress mutagenesis and may therefore represent poor standalone bactericidal targets; their partial inhibition could instead accelerate adaptive evolution and resistance emergence. Nfo is a comparatively compelling core BER candidate because *S. aureus* lacks the Xth-type redundancy present in *E. coli*, because no direct human Nfo homolog has been identified, and because structural information is available for inhibitor development. However, target essentiality under infection-relevant conditions and pharmacological vulnerability remain insufficiently established, while current inhibitor evidence is largely restricted to modeling and docking. LigA has stronger evidence for essentiality and chemical tractability but is a multifunctional DNA metabolism enzyme rather than a BER-specific target. RecJ, PolA and PolX remain biologically relevant but insufficiently validated with respect to essentiality, selective vulnerability and druggability in *S. aureus*. Thus, translation of BER-targeted strategies will require target-specific genetic validation, biochemical confirmation of inhibition, chemical-genetic analysis, and demonstration of antibacterial efficacy in infection models. The relative strengths and current limitations of the major BER-associated therapeutic targets discussed above are summarized in Table 1.

**Table 1:** Comparison of BER-associated proteins in *S. aureus* as potential antimicrobial targets: direct evidence, BER specificity, current target assessment and major limitations.

Protein	Direct evidence in *S. aureus*	Evidence type	BER specificity	Current target assessment	Main limitation
MutY	Yes	Genetic, mutagenesis under NO stress	Core BER glycosylase	Limited as standalone target	Inhibition may increase mutagenesis
MutM	Partial	Biochemical/genomic	Core BER glycosylase	Limited	Bactericidal vulnerability unproven
Nth	Limited	Mainly comparative	Core BER glycosylase	Hypothesis-generating	Direct *S. aureus* validation limited
UDG	Limited	Genomic/biochemical context; natural inhibitors	Core BER glycosylase	Conceptually interesting	Effect on viability versus mutagenesis unknown
Nfo	Yes, but incomplete	Structural/biochemical; docking	Core BER enzyme	Promising but unvalidated	Essentiality and cellular inhibition unproven
RecJ	Yes	Structural/functional	Multifunctional	Insufficient evidence	Not BER-specific
PolA	Limited	Mainly comparative	Multifunctional	Insufficient evidence	Direct *S. aureus* vulnerability unproven
PolX	Emerging	Structural/biochemical	BER-associated	Insufficient evidence	Essentiality and druggability unknown
LigA	Yes	Structural, biochemical, essentiality	Multifunctional	Chemically tractable, non-BER-specific	Broad DNA-metabolism target

## 6 Future perspectives

Although substantial progress has been made in understanding BER in *S. aureus*, several fundamental questions remain unresolved. First, much of the current mechanistic understanding of BER continues to rely on studies performed in model organisms, particularly *E. coli*, whereas direct experimental evidence for *S. aureus* remains comparatively limited. Further genetic and biochemical characterization of BER components is therefore required to define species-specific repair mechanisms.

Genome-wide approaches, including transposon sequencing (Tn-seq), CRISPR interference (CRISPRi), and chemical-genetic interaction mapping, are likely to facilitate systematic identification of essential BER components and reveal functional redundancies that cannot be detected by analysis of single-gene mutants. Such approaches may also identify synthetic lethal interactions between BER and other DNA repair pathways.

An equally important challenge is the development of selective BER inhibitors. Although structural information is now available for several BER-associated proteins, including SaNfo, LigA and PolX, most candidate inhibitors remain at the level of biochemical characterization or computational prediction. Future studies should prioritize biochemical validation, optimization of inhibitor specificity, assessment of antibacterial activity in infection models, and evaluation of synergistic combinations with conventional antibiotics and phage-based therapies.

Finally, BER should be considered within the broader context of bacterial DNA damage responses. Better understanding of the functional interplay between BER, homologous recombination, mismatch repair, nucleotide excision repair and the SOS response will be essential for predicting the consequences of BER inhibition on bacterial survival, mutagenesis and resistance evolution. Such knowledge will guide the rational design of antimicrobial strategies that maximize bacterial killing while minimizing stress-induced adaptive evolution.

## 7 Conclusion

Base excision repair plays a central role in the survival and adaptation of *S. aureus* during host infection by maintaining genome stability under oxidative and nitrosative stress. Compared with *E. coli*, the BER network of *S. aureus* appears to exhibit lower functional redundancy for several key components, making BER an attractive framework for exploring novel antimicrobial strategies. However, the therapeutic potential of individual BER proteins differs considerably and should be evaluated according to their biological function, redundancy and experimental validation.

Current evidence suggests that BER-associated proteins cannot be considered equivalent drug targets. DNA glycosylases primarily suppress oxidative mutagenesis and their inhibition may increase adaptive evolution rather than bacterial killing, whereas downstream enzymes involved in processing BER intermediates, particularly Nfo, may represent more vulnerable intervention points because of their limited redundancy. Nevertheless, most candidate inhibitors remain at the structural or biochemical stage, and their antibacterial efficacy has yet to be demonstrated *in vivo*.

Resolving these uncertainties will determine whether BER can be exploited safely and effectively without accelerating stress-induced mutagenesis and resistance evolution.
